# The cell fate regulator NUPR1 is induced by *Mycobacterium leprae* via type I interferon in human leprosy

**DOI:** 10.1371/journal.pntd.0007589

**Published:** 2019-07-25

**Authors:** Priscila R. Andrade, Manali Mehta, Jing Lu, Rosane M. B. Teles, Dennis Montoya, Phillip O. Scumpia, Euzenir Nunes Sarno, Maria Teresa Ochoa, Feiyang Ma, Matteo Pellegrini, Robert L. Modlin

**Affiliations:** 1 Division of Dermatology, David Geffen School of Medicine, University of California, Los Angeles, Los Angeles, California, United States of America; 2 Department of Molecular, Cell, and Developmental Biology, University of California, Los Angeles, Los Angeles, California, United States of America; 3 Leprosy Laboratory, Oswaldo Cruz Foundation, Rio de Janeiro, Brazil; 4 Department of Dermatology, University of Southern California School of Medicine, Los Angeles, California, United States of America; 5 Department of Microbiology, Immunology and Molecular Genetics, University of California, Los Angeles, Los Angeles, California, United States of America; EPFL, SWITZERLAND

## Abstract

The initial interaction between a microbial pathogen and the host immune response influences the outcome of the battle between the host and the foreign invader. Leprosy, caused by the obligate intracellular pathogen *Mycobacterium leprae*, provides a model to study relevant human immune responses. Previous studies have adopted a targeted approach to investigate host response to *M*. *leprae* infection, focusing on the induction of specific molecules and pathways. By measuring the host transcriptome triggered by *M*. *leprae* infection of human macrophages, we were able to detect a host gene signature 24–48 hours after infection characterized by specific innate immune pathways involving the cell fate mechanisms autophagy and apoptosis. The top upstream regulator in the *M*. *leprae*-induced gene signature was *NUPR1*, which is found in the *M*. *leprae*-induced cell fate pathways. The induction of *NUPR1* by *M*. *leprae* was dependent on the production of the type I interferon (IFN), IFN-β. Furthermore, NUPR1 mRNA and protein were upregulated in the skin lesions from patients with the multibacillary form of leprosy. Together, these data indicate that *M*. *leprae* induces a cell fate program which includes *NUPR1* as part of the host response in the progressive form of leprosy.

## Author summary

The initial interaction between a microbial pathogen and the host immune response can determine the outcome of an infection. In order to elucidate the initial events of infection by *Mycobacterium leprae*, we measured the host transcriptome of *M*. *leprae* infected human macrophages at different time points. We detected a host gene signature 24–48 hours after infection characterized by specific innate immune pathways involving the cell fate mechanisms autophagy and apoptosis. Further analysis identified *NUPR1* as the most expressed top upstream regulator in the *M*. *leprae*-induced gene signature. Our experimental data showed that *NUPR1* gene expression was dependent on the production of the type I interferon, IFN-β. Moreover, we also observed that NUPR1 was upregulated in the skin lesions from patients with the multibacillary form of leprosy. Together, our data suggests an association between the induction of *NUPR1* by *M*. *leprae* and a cell fate program that contributes to progressive mycobacterial infection in humans.

## Introduction

The causative agent of leprosy, *Mycobacterium leprae* was discovered by Armauer Hansen in 1873 and became the first bacterial pathogen to be associated with a human disease [1]. *M*. *leprae* is an intracellular pathogen that primarily infects macrophages and Schwann cells [2]. Although identified more than a century ago, it has not been possible to culture *M*. *leprae* in vitro, so bacilli are grown in the mouse foot pad and nine banded armadillos and then harvested for in vitro studies and animal experiments. Currently, there is no animal model that mimics the human disease spectrum, presenting a challenge to understand how distinct responses to the bacteria contribute to the pathogenesis of leprosy [3, 4].

Leprosy provides an excellent model to study human pathways of host defense as well as mechanisms by which an intracellular bacterium evades antimicrobial responses and establishes chronic infection. The disease presents as a clinical spectrum, with the two poles mirroring opposite immune responses to the pathogen *M*. *leprae* [5]. At one end of the spectrum, tuberculoid leprosy (T-lep) represents a self-contained form of disease, with few lesions in which bacilli are rarely found. T-lep lesions are characterized by production of Th1-cytokine expression including type II interferon (IFN-γ), known to activate macrophages to kill intracellular mycobacteria [6–8]. At the other end of the spectrum, lepromatous leprosy (L-lep) represents the disseminated form of the disease, characterized by production of Th2-cytokines that promote antibody responses but inhibit cell-mediated immunity [6–8].

In addition to the presence of distinct T cell cytokine patterns in the different forms of leprosy, there is divergence of macrophage functional programs across the spectrum of disease that can play a role in determining the host’s immune responses to the bacteria and the extent of leprosy neuropathy [7, 9, 10]. The ability of activated human macrophages to eliminate intracellular mycobacteria involves the induction of both vitamin D dependent and independent antimicrobial responses [11–15]. Activation of the vitamin D pathway leads to the induction of autophagy and antimicrobial peptides such as cathelicidin and β-defensin 2, culminating in the elimination of bacteria [6, 7, 11–13, 16]. The expression of these antimicrobial genes and the presence of cells undergoing autophagy is more prominent in T-lep than L-Lep lesions. By contrast, L-lep lesions are characterized by the accumulation of macrophages programmed to express scavenger receptors involved in phagocytosis, but lack expression of antimicrobial molecules [7]. *M*. *leprae* induces type I interferons and subsequently IL-10, all prominent in L-lep lesions. The result is inhibition of IFN-γ- and vitamin D–dependent antimicrobial responses in macrophages thereby contributing to bacterial persistence [8, 17].

The incubation time after exposure to *M*. *leprae* infection is long, months to years, such that patients with leprosy are often diagnosed after they have been chronically infected. Therefore, it is difficult to investigate the initial events in which the bacterium interacts with the host’s immune response, as is the case for *M*. *tuberculosis* [18]. Furthermore, the studies that have investigated the host macrophage response to *M*. *leprae* infection so far adopted a targeted approach, focusing on a group of molecules or pathways involved in response to infection [19–21]. Here we performed a host transcriptome analysis of *M*. *leprae* infected human monocyte-derived macrophages (MDMs) which we integrated with the transcriptomes of leprosy lesions in order to characterize the initial host-pathogen interactions that are relevant at the site of disease.

## Materials and methods

### Antibodies and cytokines

Human recombinant M-CSF (R&D Systems) was used for differentiation of blood monocytes into monocyte-derived macrophages (MDMs). Human recombinant IFN-β (BD Biosciences) and IFN-γ (BD Biosciences) were used for macrophage stimulations at the concentrations indicated. Anti-IFNAR antibody (PBL Assay Science) and corresponding isotype antibody mouse IgG2a (BioLegend) were used for neutralization experiments. Immunoperoxidase staining was performed with NUPR1 antibody (Abbexa), corresponding isotype antibody mouse IgG2b (Sigma), CD3 antibody (BD Pharmingen) and Biotinylated horse anti-mouse IgG (Vector).

### Ethics statement

Human peripheral blood was obtained from healthy donors with informed consent (UCLA Institutional Review Board #125.15.0-f). Written informed consent was provided by all study participants.

### Monocyte-derived macrophage experiments

Peripheral blood mononuclear cells (PBMCs) were isolated from peripheral blood using a Ficoll-hypaque (GE Healthcare) density gradient. MDMs were generated as previously described [15]. In brief, blood monocytes were isolated by CD14^+^ positive selection using CD14 Microbeads (Mylteni Biotec) according to the manufacturer’s instructions. For monocyte differentiation into macrophages, CD14^+^ cells were cultured for 5 days in RPMI 1640 supplemented with 10% fetal calf serum (FCS) (Omega Scientific) and M-CSF (50 ng/ml) and maintained at 37°C with 4% CO_2_. For RNA sequencing, MDMs were either uninfected and collected at 0h, or infected with *M*. *leprae* at a multiplicity of infection (MOI) of 10 for 1h, 2h, 24h and 48h. For uptake efficiency, 3 x 10^5^ MDMs were uninfected or infected with PKH26-labeled *M*. *leprae* at MOI 10 for 24 hours. Cells were then either stained with DAPI and assessed via confocal microscopy or analyzed using Flow Cytometry. For neutralization experiments, MDMs were pre-treated with anti-IFNAR blocking antibody or Isotype control (10ug/ml) one hour prior to infection with *M*. *leprae*. In a separate experiment, MDMs were stimulated with different concentrations of human recombinant IFN-β (285u/ml) *vs*. IFN-γ (1.5ng/ml) for 2h, 6h and 24h. The IFN-γ data was previously published [15]. All experiments with live *M*. *leprae* were conducted at 33°C with 4% CO_2_.

### RNA sequencing

One and a half million MDMs from one healthy donor were infected with *M*. *leprae* at multiplicity of infection (MOI) of 10 in RPMI 1640 with 10% FCS. Cells were lysed at varying time points post-infection using RLT Buffer (Qiagen) supplemented with 1% β-mercaptoethanol. Samples were added to Lysing Matrix B tubes containing 0.1mm silica beads (MP Biomedicals) and sonicated in FastPrep-24 instrument (MP Biomedicals) for 2 cycles of 45 seconds at 6.5m/s with one minute interval on ice. Total RNA was then isolated using RNeasy Micro kit (Qiagen) according to manufacturer’s protocol and quantified by RiboQuantitation and Qubit. All samples had RNA integrity above 8.0 as determined by Bioanalyzer (Agilent Technologies). Depletion of ribosomal RNA and library preparation was performed using Ribozero Gold Epidemiology (Illumina) and TruSeq Sample Preparation Kit (Illumina) as per manufacturer’s protocols. Final libraries were reassessed for quality (Qubit and Bioanalyzer), multiplexed at two samples per lane (10μM each library), and sequenced on a HiSeq 2000 sequencer (Illumina) generating 50bp single-end reads.

### Bioinformatics analysis

Sequenced reads were demultiplexed and aligned to the human reference genome hg19 (UCSC) using TopHat (version 2.0.6) and Bowtie2 (version 2.0.2). Once raw counts were generated using HTSeq, data normalization was performed using the DESeq (version 1.5) Bioconductor package. Clustering was performed on genes after filtering according to DESeq normalized counts (> 2) in any one sample and variation between maximum and minimum expression values across samples (> 2). Genes were clustered with Cluster 3.0 using single linkage and Pearson correlation as similarity measure and heatmap was generated by TreeView.

### Weighted gene network correlation analysis (WGCNA)

WGCNA was performed using the “WGCNA” R package as previously described [22]. In brief, genes were filtered first by excluding genes with 0 counts across all samples, then by calculating the overall sum of counts across all samples and removing genes in the lowest 40% quantile range. All samples were analyzed simultaneously. The function “blockwiseModules()” was used to construct signed hybrid, weighted correlation networks with a soft thresholding power of 10. Each time point was encoded as a binary vector that was one for a specific time point and zero for the other timepoints. A vector was also created for timepoint combinations including 1 + 2 hours as well as 24 + 48 hours. Module correlations were generated by coding traits (0h, 1h, 2h, 24h, 48h, 1+2h and 24+48h) as a binary matrix of zeros and ones: each sample had a value of ‘1’ for its corresponding subtype and ‘0’ for all other subtypes. The WGCNA built-in ‘Heatmap’ function was used to display the correlation and significance (*p*-value) of traits versus modules.

### Functional gene annotation analysis

Two lists of curated genes of IFN-β - and IFN-γ specific downstream genes derived from RNA-seq data from stimulated MDMs were generated (GSE125352 and GSE82227). We identified IFN-β and IFN-γ specific downstream genes by first using a 3-fold change cutoff difference between the IFNs *vs* media and including the genes that were exclusively upregulated by each cytokine. Because IFN-β and IFN-γ can induce a common set of genes, we addressed gene specificity by applying a difference of 5-fold change expression between the two stimuli. We identified 438 IFN-β specific genes and 166 IFN-γ specific genes. Tuberculoid (T-lep) and Lepromatous (L-lep) leprosy genes were derived from microarray data using molecules with *p*<0.05, fold change>2 and probe intensity average>100 as previously described [8] (GSE17763). Canonical pathway, Disease & Functions and Upstream Regulator analyses were performed using Ingenuity Pathway Analysis (IPA-Qiagen). Gene ontology (GO) enrichment analysis was performed using Cytoscape (version 3.6.0) software with ClueGO (version 2.5.0) plugin [23]. The GO term database file (updated on January 6th, 2018) was used and the significance of each term was calculated with a right-sided hypergeometric test with the Benjamini-Hochberg correction of *p*-values. Significantly overrepresented terms were defined as having Benjamini-Hochberg corrected *p*-values less than 0.05.

### Real-time Quantitative PCR

Total RNA from MDMs infected with *M*. *leprae* (MOI 10) or stimulated with different concentration of human recombinant IFN-β was obtained using TRIzol reagent (Invitrogen) and cDNA was prepared using iScript cDNA Synthesis Kit (Bio-Rad Laboratories) according to the manufacturer’s instructions. RT- qPCR was performed using KAPA SYBR FAST qPCR kit (KAPA Biosystems) and normalized to reference gene *36B4* (NM_001002) (Forward primer: 5′-CCA CGC TGC TGA ACA TGC T -3′ and Reverse primer: 5′-TCG AAC ACC TGC TGG ATG AC -3′). Arbitrary units were calculated using the 2^-(ΔΔCt)^ method [11]. *NUPR1* (NM_001042483, NM_012385) primer set was obtained from Quantitect (Qiagen) (QT00088382). Experiments were performed using the CFX96 touch real time PCR detection system (Bio-Rad Laboratories).

### Leprosy biopsy specimens and immunoperoxidase labeling

Skin biopsy specimens were collected from untreated patients at the Leprosy Clinic at the Oswaldo Cruz Foundation in Brazil as well as at the Hansen’s Disease Clinic at Los Angeles Country and University of Southern California Medical Center. The diagnosis and classification of patients were determined based on clinical and histopathological criteria of Ridley and Jopling [5]. Cryosections (4μm) from skin lesions of T-lep and L-lep patients were incubated with normal horse serum followed by staining with anti-NUPR1, anti-CD3, or isotype control. Sections were subsequently incubated with biotinylated horse anti-mouse IgG, ABC Elite system, and AEC Peroxidase Substrate Kit (Vector Laboratories) and counterstained with hematoxylin prior to mounting in crystal mounting medium (Biomeda). NUPR1 and CD3 staining was visualized using a Leica microscope (Leica 250). NUPR1 staining was quantified using ImmunoRatio [24].

### Live *Mycobacterium leprae*

Live *M*. *leprae* (unlabeled or labeled with PKH26) was graciously provided by Dr. Ramanuj Lahiri of the National Hansen’s Disease Program, Health Resources Service Administration, Baton Rouge, Louisiana. *M*. *leprae* was grown in athymic (nu/nu) mouse foot pad as previously described [25].

### Statistical analysis

Descriptive statistics were calculated for all continuous variables. Specifically, the mean ± the standard error of the mean (SEM) were reported for normally distributed data. Data distribution was graphically assessed by using quantile-quantile (Q-Q) plots. The repeated measures one way-ANOVA with Bonferroni’s multiple comparisons test was used to evaluate differences among three or more groups that satisfied the normality assumption. The Geisser-Greenhouse correction was applied when the sphericity assumption was not met.

Repeated measures two-way ANOVA with Bonferroni’s multiple comparisons test was performed to evaluate differences involving two variables among groups that satisfied the normality assumption. Comparisons between two independent groups with normal distribution were performed using the two-sample T-test when equal variance was assumed or the Welch’s T-test when unequal variances were observed. Enrichment analyses of the overlap between IFNs or leprosy lesion gene signatures and the *M*. *leprae* transcriptome were performed using the hypergeometric distribution to control for differences in the overall number of differentially expressed genes. The hypergeometric distribution (hypergeometric test) is equivalent to the one-tailed version of Fisher’s exact test and determines the degree the observed amount of enrichment is greater than expected [26, 27]. The fold change was calculated to display both over and under enrichment of the gene sets in the *M*. *leprae* gene induced signature as previously described [8]. The over enrichment fold change was calculated as number of Observed genes/number of Expected genes. However, in order to avoid plotting fold changes < 1, we calculated the under-enrichment fold changes as -1(number of Expected genes/number of Observed genes). All statistical analyses were performed using GraphPad Prism 7 software. All tests except the hypergeometric test were two-sided, and the level of statistical significance was set at 0.05.

## Results

### Pathway Analysis of *M*. *leprae*-induced gene signatures in human MDMs

To investigate the effect of *M*. *leprae* on the innate immune response in human macrophages, we performed RNA sequencing (RNA-seq) on infected cells in vitro. Briefly, CD14^+^ monocytes were isolated from a single healthy donor and cultured with recombinant human M-CSF for five days to allow differentiation into macrophages (S1 Fig). The monocyte-derived macrophages (MDMs) were infected with *M*. *leprae* at a multiplicity of infection (MOI) of 10 for 1, 2, 24 and 48 hours, as we found consistent with previous studies [8, 17]. This resulted in infection of over 85% of the cultured macrophages (S2 Fig). RNA was harvested at each time point, yielding five samples that were subsequently sequenced (S3 Fig).

**Fig 1:**
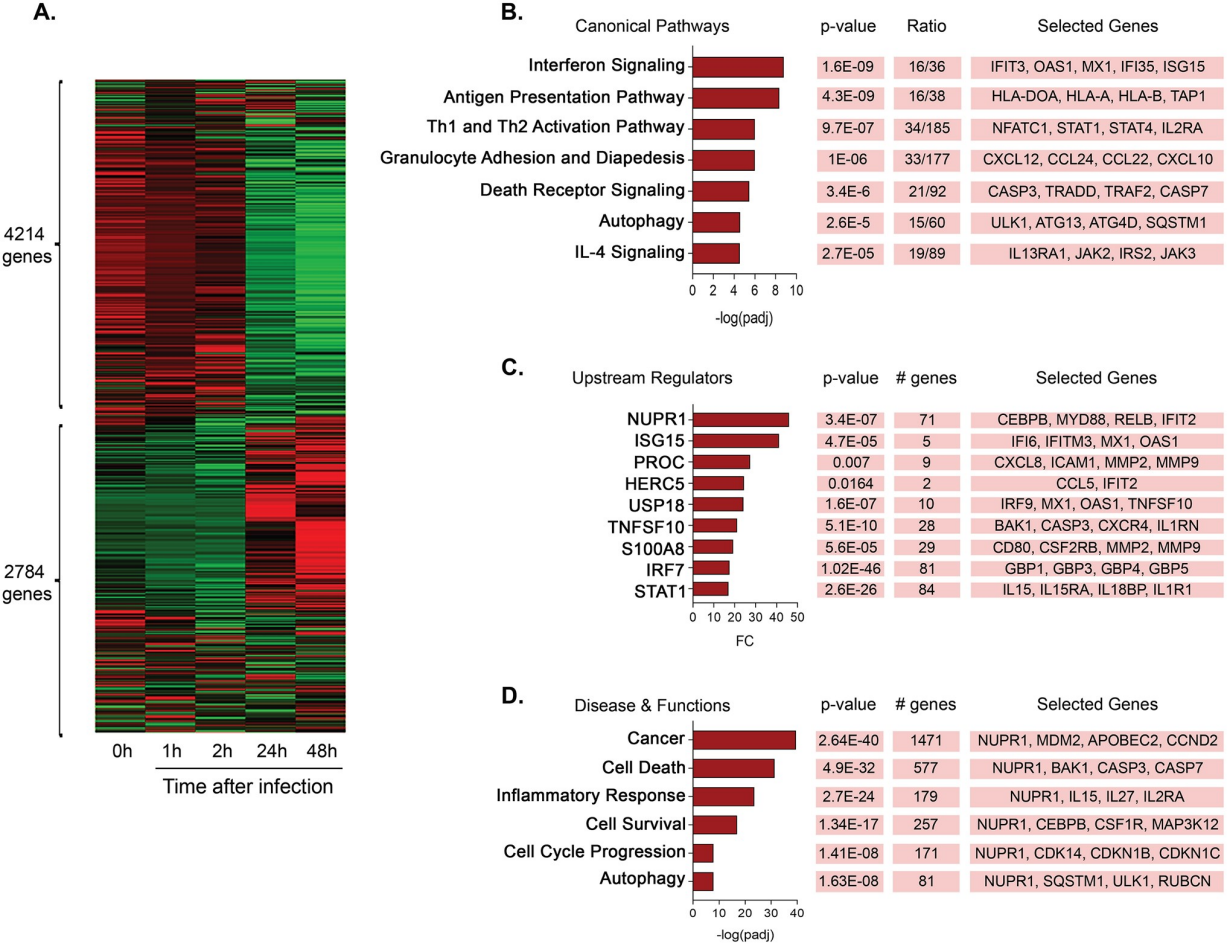
*M*. *leprae* infection leads to induction of cell fate pathways in MDMs. **(A)**. Heatmap of clustered genes induced by *M*. *leprae* at different time points. Color green indicates downregulated genes and color red indicates upregulated genes. **(B)**. Top Canonical Pathways significantly enriched in the *M*. *leprae* induced-gene signature by IPA core analysis. **(C)**. Most expressed upstream regulators by fold-change (FC) in the *M*. *leprae* induced-gene signature. **(D)**. IPA Disease and Functions analysis of the *M*. *leprae* induced-gene signature. The *p*-value is calculated by Fisher’s Exact Test and measures the significant overlap between the dataset genes and the genes that belong to a canonical pathway, upstream regulator or the ‘Disease and Function’ categories in the IPA knowledge database. Adjusted *p*-values (padj) were calculated using Bonferroni correction. Ratios represent the number of genes in the dataset that appear in an IPA term divided by the total number of genes of the same term. Genes of the canonical pathway, upstream regulator or ‘Disease and Functions’ analyses were selected based on their functional relevance and displayed in boxes. # genes represent the exact number of molecules in our dataset regulated by an upstream regulator or found in the Disease and Functions categories.

Gene clustering analysis identified a cluster of 4,214 genes detected at either 0h (uninfected), or 1 and 2 hours after infection. We also observed a cluster of 2,784 late response genes differentially expressed at 24 and 48h post-infection (Fig 1A), of which 2,107 were upregulated by >1.5-fold change (FC) (S1 Table). In order to determine the biological functions of this *M*. *leprae* induced gene signature, we performed Ingenuity Pathway Analysis using their curated database of canonical pathways. The most significantly enriched canonical pathways in the *M*. *leprae* induced gene signature were “Interferon Signaling” (-log_10_ *p*-value = 8.78), “Antigen Presentation Pathway” (-log_10_ *p*-value = 8.36), and “Th1 and Th2 activation pathway” (-log_10_ *p*-value = 6.01 (Fig 1B; S2 Table). We noted that there was significant enrichment for cell fate pathways including “Death Receptor Signaling” (-log_10_ *p*-value = 5.46) and “Autophagy” (-log_10_ *p*-value = 4.57) in the *M*. *leprae* induced gene set. The “Death Receptor Signaling” pathway included *CASP3*, *CASP7* and *TRADD*, which are involved in apoptosis, and the “Autophagy” pathway included the genes *ATG13*, *ATG4D* and *ULK1*, which play a role in autophagosome formation.

We also found enrichment of cell fate pathways in the *M*. *leprae* induced transcriptome using a second method, weighted gene correlation network analysis (WGCNA), which is an unbiased approach that defines modules of highly interconnected genes based on pairwise correlations using only the gene expression data without any identifiers [22]. The individual modules were next correlated with the expression data at specific time points after *M*. *leprae* infection, identifying six modules significantly associated with defined time points (*p*<0.05, correlation>0.8) (S4 Fig; S1 Table). ‘GreenYellow’ (1,627 genes) was the only module that correlated with the combined 24+48h vector (r = 0.99, *p* = 6x10^-4^). We observed that 883 of the 1,627 genes in the ‘GreenYellow’ module were also upregulated in the *M*. *leprae* induced signature (enrichment *p*-value = 6.15E^−611^; Fold-change enrichment = 7.2) (S4 Fig). Ingenuity analysis of the 1,627 genes in this module identified similar canonical pathways as the *M*. *leprae* induced signature, despite the relatively small number of datasets used to generate the WGCNA [22] (S5 Fig, S2 Table). The canonical pathways identified in ‘GreenYellow’ included “Interferon Signaling” (-log_10_ *p*-value = 7.8), “Antigen Presentation Pathway” (-log_10_ *p*-value = 6.7), and “Autophagy” (-log_10_ *p*-value = 2.98).

### *NUPR1* is a top upstream regulator of the *M*. *leprae* induced gene signature

We performed further investigation by Ingenuity Pathway Analysis to identify upstream regulators that could be driving the *M*. *leprae*-induced gene signature. We filtered this analysis based on FC>1.5 for expression of the upstream regulator gene in the *M*. *leprae* induced gene signature and included only the transcription and translational regulators, cytokines and enzymes categories of the IPA database. Nuclear protein 1 (*NUPR1*) was the most induced upstream regulator in the signature (Fold change = 45.9) (Fig 1C, S2 Table). Type I interferons (IFNs), specifically *IFNA2* were also identified as upstream regulators, although *IFNA2* expression was not induced (FC>1.5) by *M*. *leprae*. However, three *M*. *leprae* induced upstream regulators are known to be induced by type I IFN: *ISG15* (FC = 41.1), *HERC5* (FC = 24.4) and *USP18* (FC = 24.2). Furthermore, two other interferon signaling molecules were identified as upstream regulators, *STAT1* (FC = 16.9) and *IRF7* (FC = 17.5), and their downstream target molecules were also induced by *M*. *leprae*.

Further analysis of *M*. *leprae*-induced gene signature revealed enrichment of biological functions such as ‘Cell Death’, ‘Cell Survival’, ‘Inflammatory Response’ and ‘Autophagy’ (Fig 1D; S2 Table). We noted that *NUPR1* was present in all of these biological functional pathways, consistent with its known role in regulating cell fate.

### *M*. *leprae* induction of *NUPR1* is dependent on type I IFN signaling

**Fig 2:**
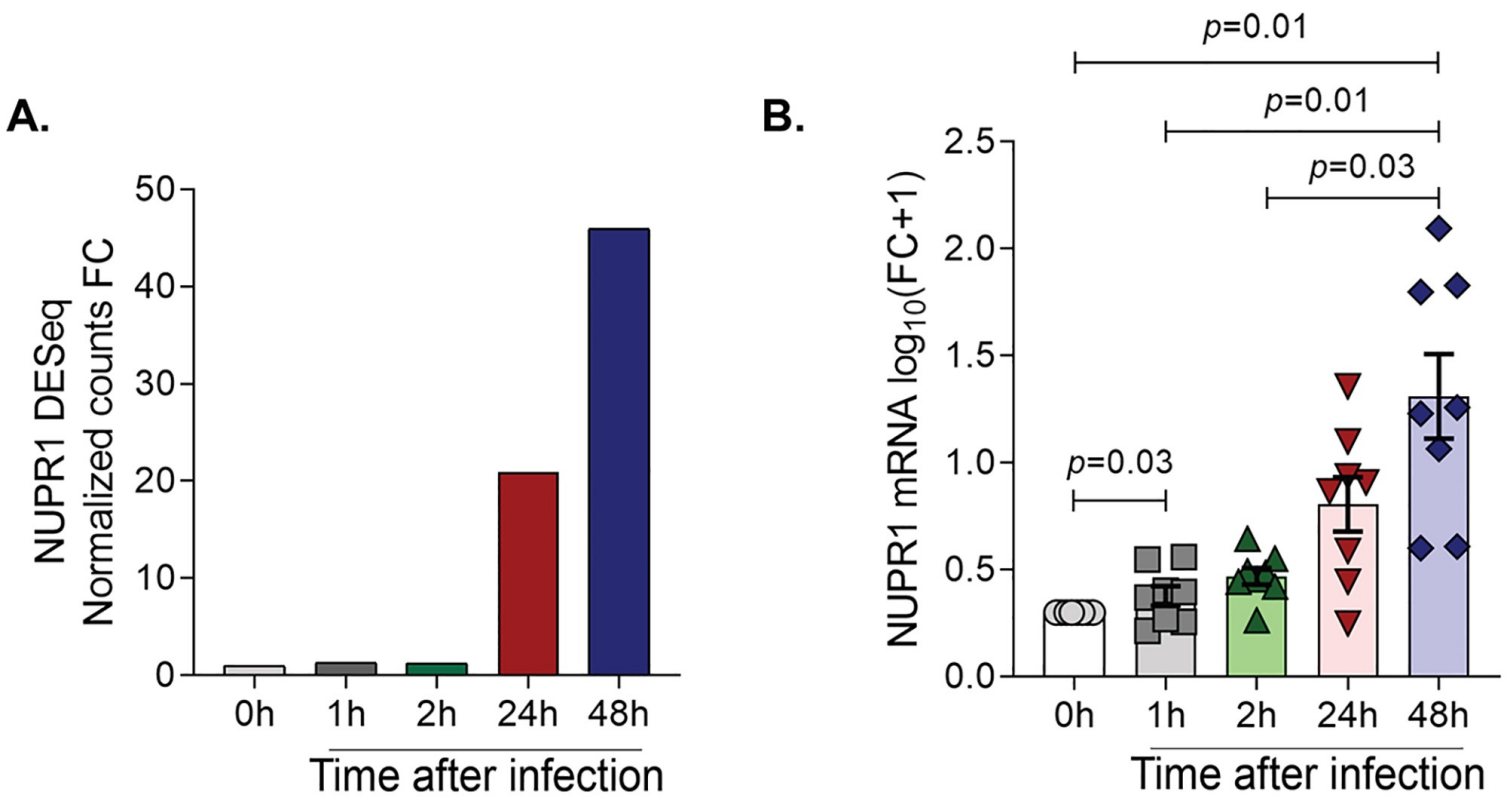
*M*. *leprae* infection induces *NUPR1* gene expression in MDMs. **(A)**. *NUPR1* DESeq normalized counts fold change at different time points post-*M*. *leprae* infection in MDMs. **(B)**. *NUPR1* gene expression measured by qPCR in *M*. *leprae* infected MDMs at different time points (n = 8). Statistical analyses were performed using repeated measures one way-ANOVA with the Geisser-Greenhouse correction followed by Bonferroni’s multiple comparisons test.

Given that *M*. *leprae* infection of MDMs induced *NUPR1* mRNA at 24h and 48h in the RNA-seq data (Fig 2A), we further validated this finding by qPCR in eight additional donors. *NUPR1* mRNA was upregulated by a mean log_10_ fold change of 1.3 at 48h in MDMs post-infection compared to uninfected cells (Fig 2B).

**Fig 3:**
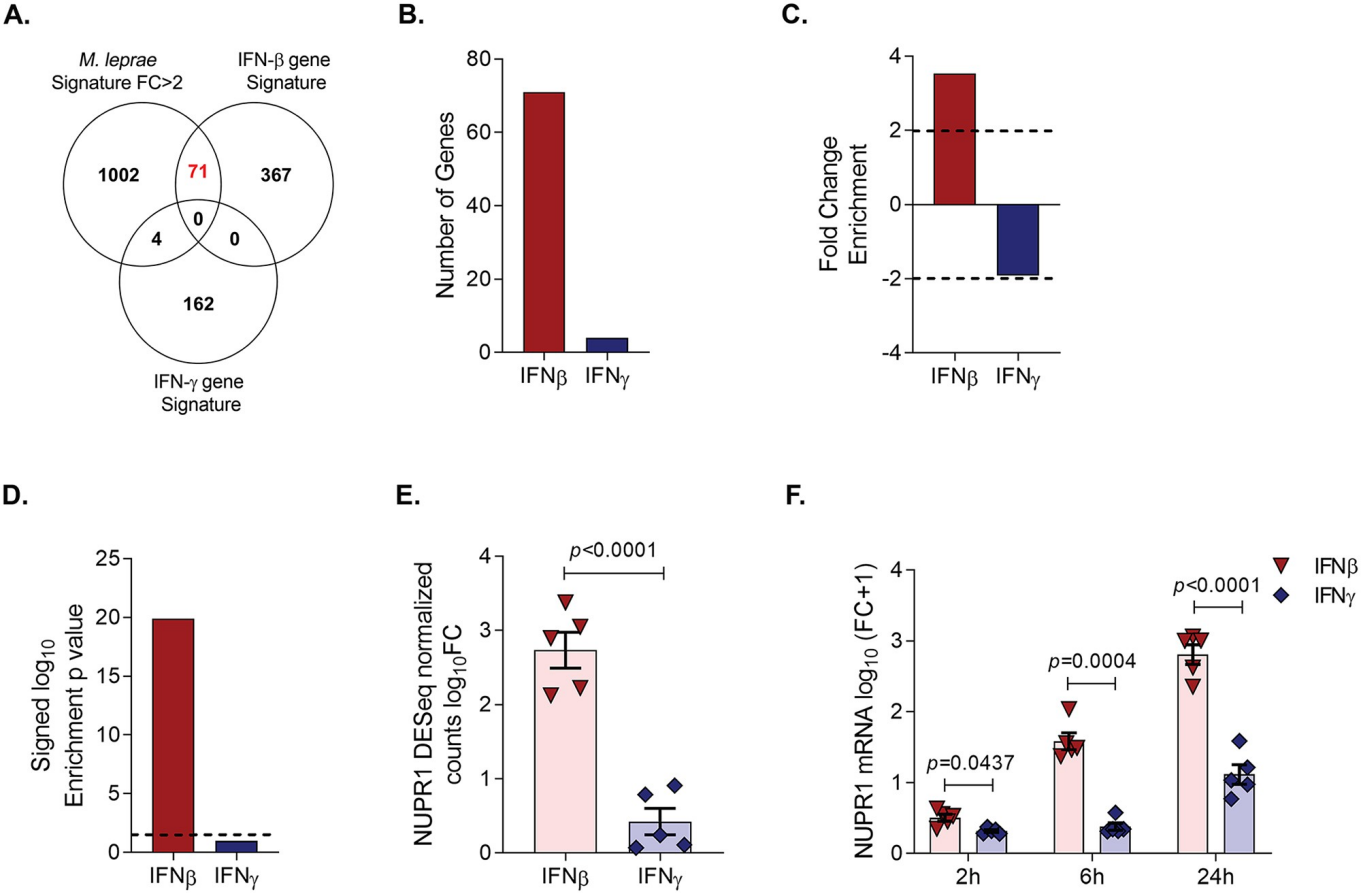
*NUPR1* gene expression is induced by *M*. *leprae* and type I interferon. **(A)**. Overlap of the *M*. *leprae* induced gene signature (fold change >2) with IFN-β and IFN-γ specific gene signatures from cytokine-stimulated MDMs. **(B)**. Number of IFN-β and IFN-γ specific genes found in the *M*. *leprae* induced gene signature. **(C)**. Fold change enrichment (see Materials and methods) of IFN-β and IFN-γ specific genes found in the *M*. *leprae* induced gene signature. **(D)**. -log_10_ enrichment *p*-value of IFN-β and IFN-γ specific genes found in the *M*. *leprae* induced gene signature calculated by hypergeometric test. **(E)**. *NUPR1* fold change of DESeq normalized counts from RNA-seq data of MDMs stimulated with IFN-β and IFN-γ (n = 5) at 24h. **(F)**. *NUPR1* gene expression fold change in IFN-β (285 u/ml) and IFN-γ (1.5 ng/ml) stimulated MDMs measured by qPCR at 2, 6 and 24h (n = 5). Statistical analyses were performed using the Two-Sample T test **(E)** and repeated measures two way-ANOVA with the Geisser-Greenhouse correction followed by Bonferroni’s multiple comparisons test **(F)**.

Given that “Interferon Signaling” was identified in the *M*. *leprae* induced gene signature, we sought to determine whether this reflected a type I- and/or type II IFN response. Considering that expression of the genes encoding the type I and type II IFNs was not detected in the *M*. *leprae* induced gene signature, we utilized an integrative bioinformatics approach to attribute the relative contribution of the type I and type II IFNs to the *M*. *leprae* induced immune response. The genes with fold change >2 in the *M*. *leprae* induced gene signature were overlapped with curated lists of IFN-β- and IFN-γ specific downstream genes derived from RNA-seq data from interferon-stimulated MDMs (GSE125352 and GSE82227). We found an enrichment of IFN-β specific genes 3.53-fold higher than expected in the *M*. *leprae* induced gene signature (-log_10_ *p*-value = 19.9), with 71 genes found in this overlap, including *NUPR1* (Fig 3A–3D; S3 Table). By contrast, there were only four genes in common with the IFN-γ-specific dataset, suggesting a predominance of type I IFN downstream genes, which is characteristic of chronic mycobacterial infections including tuberculosis and leprosy [8, 28].

Although *NUPR1* was identified as an IFN-β-specific gene in the *M*. *leprae* gene signature, it had not been previously shown that this gene is induced by type I IFNs. We assessed the ability of type I and type II IFNs to induce *NUPR1* gene expression by mining the IFN-β- and IFN-γ-stimulated MDM RNA-seq data derived from five donors. *NUPR1* mRNA was highly induced by IFN-β, increasing by a mean log_10_ fold change of 2.73, but only modestly induced by IFN-γ (mean log_10_ fold change = 0.42) (GSE125352 and GSE82227) (Fig 3E). These results were validated by qPCR in the same five donors, which confirmed that IFN-β strongly induced NUPR1 at 24h (Fig 3F).

**Fig 4:**
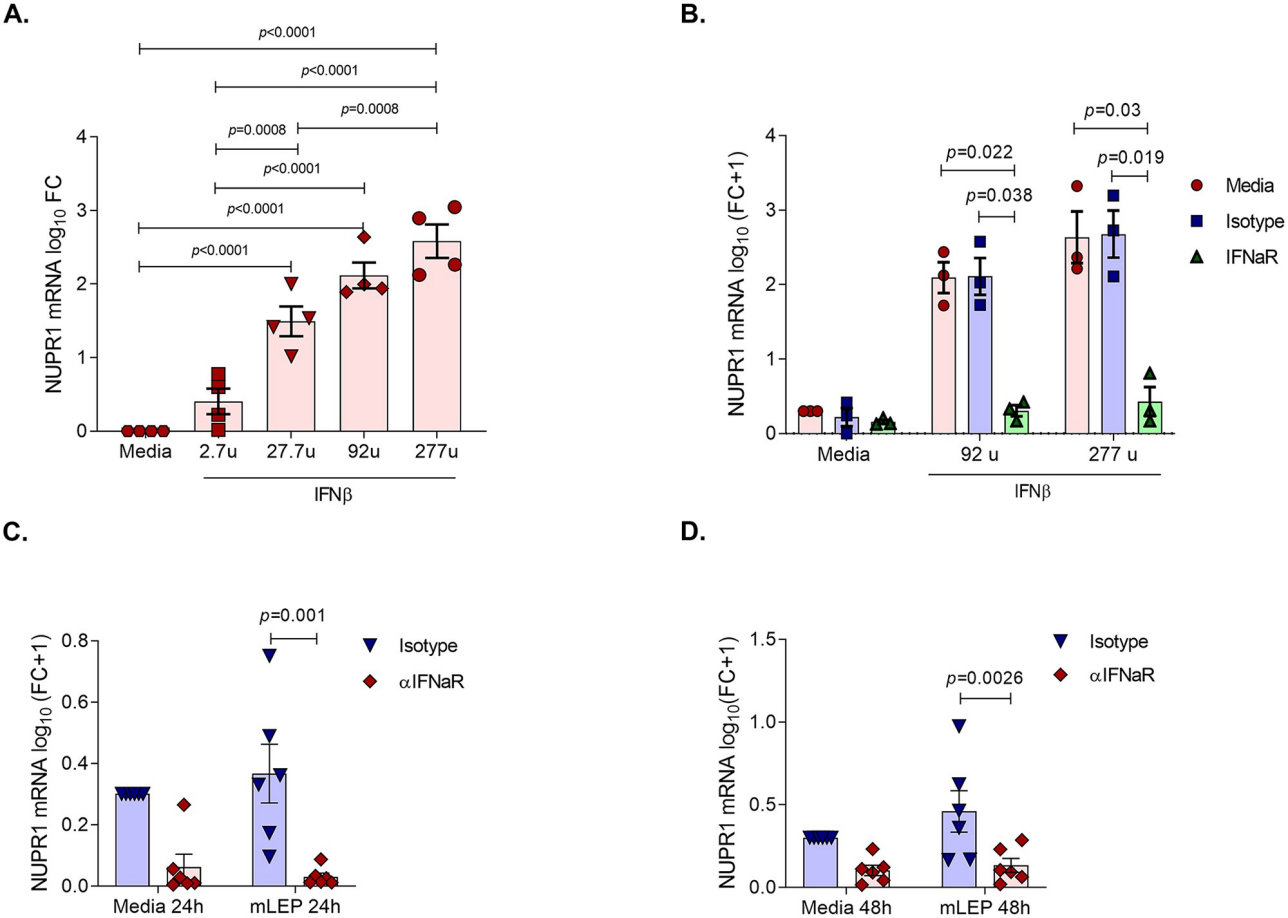
*M*. *leprae* induction of *NUPR1* is dependent on type I IFN signaling. **(A)**. Effect of different doses of IFN-β on the induction of *NUPR1* gene expression in MDMs measured by qPCR (n = 4). **(B)**. Effect of IFNAR blocking with different doses of IFN-β on *NUPR1* gene expression in MDMs measured by qPCR (n = 3). **(C)** and **(D)**. Evaluation of the effect of *M*. *leprae* infection on *NUPR1* gene expression during blockage of IFN-β signaling at 24 and 48h measured by qPCR (n = 6). Statistical analyses were performed using repeated measures one way-ANOVA **(A)**, repeated measures two way-ANOVA with the Geisser-Greenhouse correction **(B)** and repeated measures two way-ANOVA **(C** and **D)** followed by Bonferroni’s multiple comparisons test **(A-D)**.

To establish whether the induction of *NUPR1* in MDMs by *M*. *leprae* infection involved type I IFN signaling, we first performed a dose titration in four additional donors. This analysis found that the ability of IFN-β to induce *NUPR1* gene expression was dose dependent (Fig 4A). As expected, IFN-β induction of *NUPR1* in MDMs was effectively blocked by αIFNAR antibody (Fig 4B). Next, to evaluate if *M*. *leprae* induction of *NUPR1* gene expression was dependent on IFN-β signaling, we incubated MDMs with αIFNAR antibody prior to infection and, in six donors, observed a drastic decrease in *NUPR1* expression at 24 and 48h (Fig 4C and 4D). These data indicate that *M*. *leprae* induction of *NUPR1* is dependent on the activity of type I IFN.

### *NUPR1* protein is more highly expressed in L-lep lesions *versus* T-lep lesions

**Fig 5:**
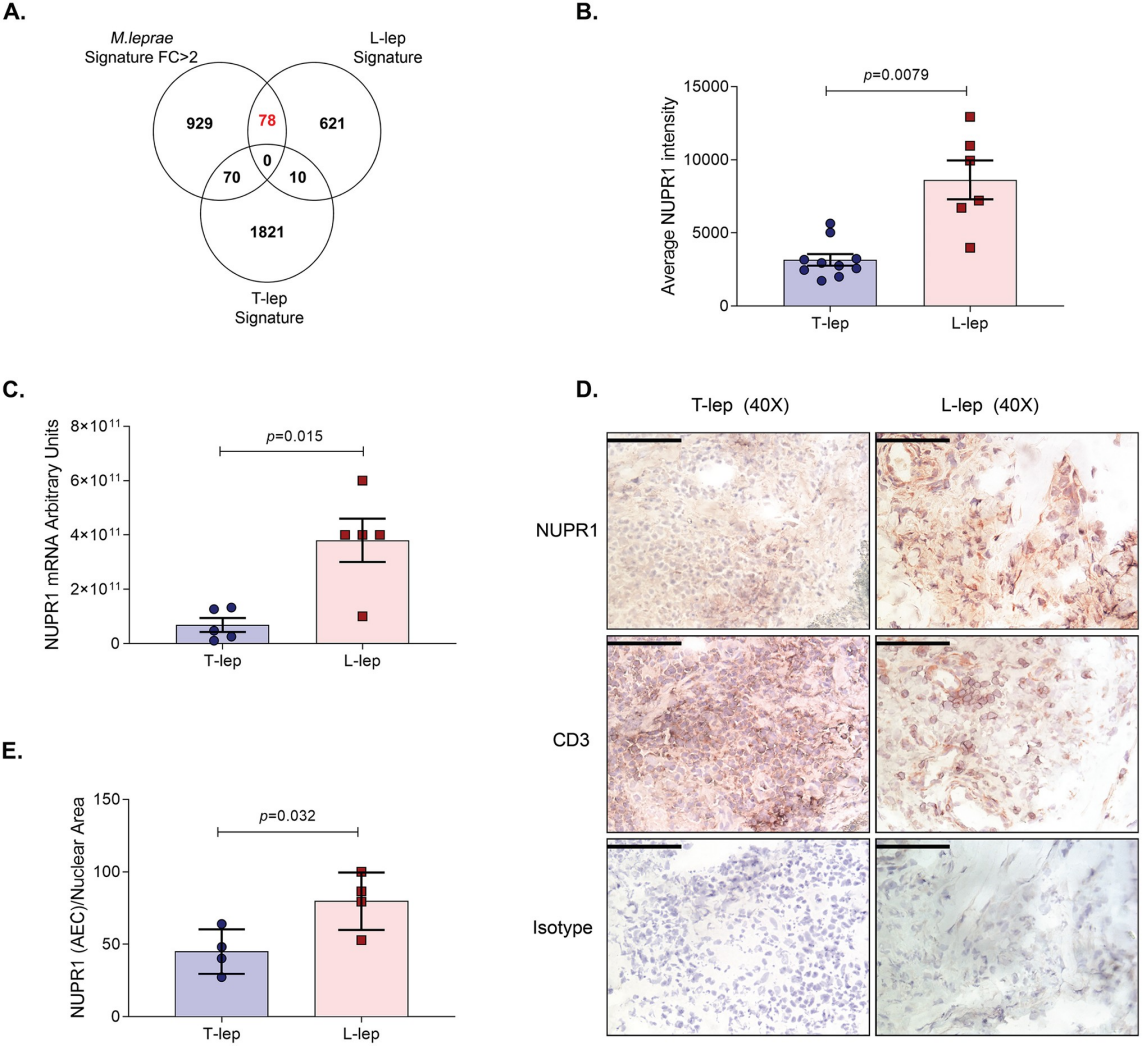
NUPR1 is highly expressed in L-lep skin lesions. **(A)**. Overlap of *M*. *leprae* induced gene signature (Fold change >2) with lepromatous (L-lep) and tuberculoid (T-lep) skin lesion specific gene signatures (FC>2; *p*<0.05; probe intensity average>100) from leprosy skin lesion microarray data [8]. **(B)**. *NUPR1* normalized probe intensity in leprosy lesion microarray data in L-lep and T-lep samples. **(C)**. *NUPR1* gene expression in L-lep and T-lep skin lesions measured by qPCR (n = 5). **(D)**. *NUPR1* protein expression in L-lep (n = 4) and T-lep (n = 4) skin lesions measured by immunohistochemistry. Scale bars (50μm), original magnification 400x. **(E)**. Quantification of NUPR1 staining in L-lep and T-lep skin lesions by ImmunoRatio. Statistical analyses were performed using the Welch’s T test **(B** and **C)** and the Two-sample t-test **(E)**.

To link the *M*. *leprae* induced gene signature in human macrophages with gene expression in leprosy lesions, we overlapped the genes with fold change >2 in the *M*. *leprae* induced gene signature with the T-lep and L-lep lesion transcriptome signatures previously published (FC>2, *p*<0.05, probe intensity average >100) (GSE17763) [8]. There was a significant 2.4-fold enrichment of L-lep genes in the *M*. *leprae* induced gene signature in MDMs (-log_10_ *p-value* = 12.1) and *NUPR1* was identified in the overlap (Fig 5A, S3 Table, S6 Fig). *NUPR1* gene expression was 2.7-fold greater in L-lep versus T-lep lesions in the microarray data (Fig 5B) (GSE17763) [8]. We confirmed *NUPR1* expression in leprosy lesions by qPCR of additional five T-lep and five L-lep samples and detected a 5.5-fold greater expression in L-lep vs. T-lep specimens (Fig 5C). Next, NUPR1 protein expression in leprosy skin lesions was evaluated by immunoperoxidase staining and concordantly, NUPR1 was more abundant in L-lep versus T-lep lesions (Fig 5D). In L-lep granulomas, NUPR1 was expressed in the nuclear, perinuclear and cytoplasmic compartments of large cells with an ovoid nucleus resembling macrophages. In contrast, CD3 was detected on the membrane of small round cells resembling lymphocytes in both L-lep and T-lep lesions. Using the online software ImmunoRatio [24], we quantified the overlap of NUPR1 immunoperoxidase staining with hematoxylin-stained nuclei and observed that ~75% of cells in L-lep lesions were positive for NUPR1 versus ~45% of cells in T-lep lesions (Fig 5E). These data suggest that *NUPR1* is induced in the early stages of *M*. *leprae* infection as well as differentially expressed at the site of disease in lepromatous leprosy.

## Discussion

Uncovering the initial events by which an intracellular bacterium interacts with the host immune response is essential for delineating pathways of host defense as well as strategies by which the pathogen evades or alters them to establish a chronic infection. Given that *M*. *leprae* is an obligate intracellular pathogen, we investigated the initial interaction between *M*. *leprae* and macrophages, using MDMs as an in vitro model and measuring the host transcriptome induced by *M*. *leprae*. Our data revealed that *M*. *leprae* induces a host gene signature at 24–48 hours after infection characterized by specific innate immune pathways involving cell fate mechanisms including autophagy and apoptosis. We observed that the most expressed upstream regulator in the *M*. *leprae* induced gene signature was *NUPR1*, which is part of the cell fate pathways, and demonstrated that its induction by *M*. *leprae* was dependent on the activity of type I IFN. The differential expression of *NUPR1* in skin lesions from patients with multibacillary infection suggests an association between the induction of *NUPR1* by *M*. *leprae* and a cell fate program that contributes to progressive mycobacterial infection in humans.

Functional pathway analyses of the *M*. *leprae* induced gene signature in MDMs revealed that *NUPR1* was the highest induced upstream regulator of this gene set. Our bioinformatics analysis indicated that *NUPR1* was part of an IFN-β–induced gene signature that overlapped with the *M*. *leprae* induced gene signature. Although we initially found that *M*. *leprae* infection of macrophages induced *NUPR1* mRNA by transcriptome analysis, we validated this finding by RT-qPCR in additional donors. We provide novel evidence that IFN-β induced *NUPR1* mRNA and determined that the ability of *M*. *leprae* to induce *NUPR1* was dependent on the activation of the type I IFN receptor. Finally, the overexpression of *NUPR1* in lesions from the progressive forms of leprosy was validated by RT-qPCR and immunohistochemistry in skin biopsy samples, consistent with the finding that IFN-β induced genes are significantly enriched in L-lep lesions [8]. The overexpression of type I IFN downstream genes has also been linked to the pathogenesis of active disease in tuberculosis [28]. In vitro studies have shown that mycobacterial infection can lead to release of mitochondrial and bacterial DNA, as well as generation of cyclic di-nucleotides that lead to activation of cyclic GMP-AMP Synthase (cGAS) and Stimulator of Interferon Genes (STING), culminating in the induction of type I IFN gene expression [29–34].

Analysis of the *M*. *leprae* induced macrophage transcriptome identified cell fate pathways involved in both autophagy and apoptosis. Both pathways represent host mechanisms to deal with cellular damage. Autophagy results in sequestration and degradation of damaged organelles and proteins towards cell preservation, whereas, apoptosis is a response to cell damage that results in programmed cell death. These pathways are generally cross-inhibitory, such that a given cell undergoes either autophagy or apoptosis, but in some instances, autophagy can induce apoptosis [35, 36]. The two pathways can also collaborate to maintain tissue homeostasis, such that autophagy provides a mechanism for clearing apoptotic cellular debris [37]. In leprosy lesions, there is a greater frequency of cells undergoing autophagy as well as apoptosis in T-lep compared to L-lep patients [21, 38, 39]. Autophagy has an important role in controlling mycobacterial infection, required for the vitamin D induction of antimicrobial activity against mycobacteria [11, 12, 16, 40, 41], although one component of the autophagy machinery, autophagy protein 5 (*ATG5*), may contribute to host defense via an autophagy-independent pathway suggesting that other mechanisms may be involved [42]. Therefore, the induction of genes involved in both autophagy and apoptosis by *M*. *leprae* may represent a host response to kill the bacteria and clear damaged cells.

*NUPR1* is a multifunctional protein capable of interacting with a great variety of molecules, and thus regulating several intracellular pathways involved in cell fate and stress responses [43]. IPA ‘Disease & Function’ annotation of the *M*. *leprae* induced signature showed that *NUPR1* was involved in autophagy and cell death pathways. There is experimental evidence to suggest that *NUPR1* contributes to the inhibition of autophagy and apoptosis [44–48], with recent studies showing that *NUPR1* knockdown leads to induction of apoptosis [49–52]. However, information on the role of *NUPR1* in these processes is conflicting and seem to be determined by the cell metabolic and environmental context [44–47, 53–57]. Nevertheless, the expression of *NUPR1* was greater in L-lep vs. T-lep lesions, inversely correlating with the reported frequency of autophagy and apoptosis [21, 38, 39]. Impairment of autophagic flux was observed in macrophages in multibacillary L-lep skin lesions [21] and *M*. *leprae* was shown to inhibit autophagy by the induction of *OASL* gene expression [58]. *OASL* is induced by type I IFN and contributes to the downregulation of the antimicrobial peptide cathelicidin [58]. Furthermore, some alleles of *PARK2* gene, a ubiquitin ligase involved in ubiquitin-mediated autophagy of mycobacteria, are associated with leprosy susceptibility [59, 60]. Apoptosis has also been detected more frequently in the T-lep skin lesions when compared to L-lep specimens [38, 39, 61], which can be associated with higher expression of the anti-apoptotic molecule BCL-2 in lepromatous skin lesions [39, 61].

*NUPR1* was found to be upregulated in the host response to infection by *Histoplasma capsulatum* [62] and detected as an upstream regulator in the host transcriptome associated with other fungal and bacterial infections [63–65]. Although our study was exploratory, it did result in novel findings: 1) We report for the first time that *NUPR1* is expressed at the site of infectious disease; and, 2) We provide novel information about the mechanism of *NUPR1* induction, demonstrating that it is induced by a human pathogen via the production of type I IFN and giving new insight into the link between *NUPR1* and microbial infection. It remains to be determined how upregulation of *NUPR1* affects the fate of *M*. *leprae* infected macrophages. The identification of such pathways that favors the survival and persistence of *M*. *leprae* within host cells [6, 8, 17, 38, 39, 61] is needed to identify potential host-directed therapy targets to regulate cellular homeostasis as part of the host immune response against infection by *M*. *leprae*.

## Supporting information

S1 Fig Experimental Design Diagram of *M*. *leprae*-infected human MDMs RNA sequencing. Human monocytes were obtained from a heathy donor and cultured for 5 days with MCSF to differentiate into monocyte-derived macrophages (MDMs). MDMs were infected with live *M*. *leprae* and RNA was harvested at 1, 2, 24 and 48h post-infection and prepared for RNA sequencing. Uninfected control is referenced as 0h.
(TIF)

S2 Fig Efficiency of *M*. *leprae* uptake by MDMs. MDMs were infected with PKH26-labeled *M*. *leprae* (red) at MOI 10 and uptake was assessed via confocal microscopy (left) and flow cytometry (right). Nuclei are stained with DAPI (blue) (n = 1).
(TIF)

S3 Fig RNA sequencing coverage. Total number of RNA sequencing reads obtained for the individual sample at 0h (uninfected) and 1h, 2h, 24h and 48h post-*M*. *leprae* infection.
(TIF)

S4 Fig WGCNA of *M*. *leprae* induced gene signature. **(A)**. Signed WGCNA of log2RPKM expression values from genes induced by *M*. *leprae* at different time points. Correlation of time points (x-axis) to WGCNA module eigengenes (y-axis) are displayed as a heatmap. The *p-*values (bottom) for each *r* correlation value (top) are indicated for each module and each time point. Red indicates positive correlation and green indicates an inverse correlation. **(B)**. -log_10_ enrichment *p*-value of *M*. *leprae* induced genes at 24+48 hours found in the WGCNA modules calculated by hypergeometric test. **(C-H)**. Venn Diagrams depicting overlap between *M*. *leprae* induced gene signature and WGCNA modules significantly correlated with infection time points.
(TIF)

S5 Fig Bioinformatics analysis of gene networks derived from *M*. *leprae* infected MDMs. Ingenuity Pathway Analysis (IPA) was performed on the WGCNA modules with a significant positive correlation (r>0.8; *p*<0.05) with infection time points. **(A)**. ‘GreenYellow’ module correlated with the 24 + 48h vector. **(B)**. ‘Darkturquoise’ module correlated with the 24h time point. **(C)**. ‘AntiqueWhite4’ module correlated with 48h. **(D)**. ‘DarkGreen’ module correlated with the 48h time point. **(E)**. ‘SkyBlue’ module correlated with the 48h time point. IPA core analyses display the Canonical Pathways significantly overrepresented in each module. The *p*‐value is calculated by Fisher’s Exact Test and measures the significant overlap between the dataset genes and the genes that belong to a canonical pathway in the IPA knowledge database. Adjusted *p*-values (padj) were calculated using Bonferroni correction. Ratios represent the number of genes in the module that appear in the canonical pathway divided by the total number of genes in that specific canonical pathway. Selected genes of each canonical pathway are displayed based on their functional relevance.
(TIF)

S6 Fig *M*. *leprae*-induced genes are enriched in L-lep gene signature. Hypergeometric enrichment analysis of overlap of *M*. *leprae*-induced gene signature (fold change >2) with the most expressed genes in L-lep and T-lep lesions (FC>2; *p*<0.05; probe intensity average>100). **(A)**. Number of genes of the T-lep and L-lep gene signature found in the *M*. *leprae-*induced gene signature. **(B)**. Fold change enrichment (see Materials and methods) of T-lep and L-lep genes in the *M*. *leprae-*induced gene signature. **(C)**. -log_10_ enrichment *p*-value of T-lep and L-lep genes found in the *M*. *leprae* induced gene signature calculated by hypergeometric test.
(TIF)

S1 Table *M*. *leprae* induced gene signature and WGCNA modules. (XLSX)

S2 Table Ingenuity Pathway Analysis of *M*. *leprae* induced gene signature and WGCNA modules. (XLSX)

S3 Table Type I IFN and L-lep gene enrichment analysis in *M*. *leprae* induced gene signature. (XLSX)
