# Supplementary figures and images for: The cell fate regulator NUPR1 is induced by Mycobacterium leprae via type I interferon in human leprosy
Source: PLoS Negl Trop Dis. 2019 Jul 25;13(7):e0007589. doi: 10.1371/journal.pntd.0007589 (PMC6684084; doi:10.1371/journal.pntd.0007589)

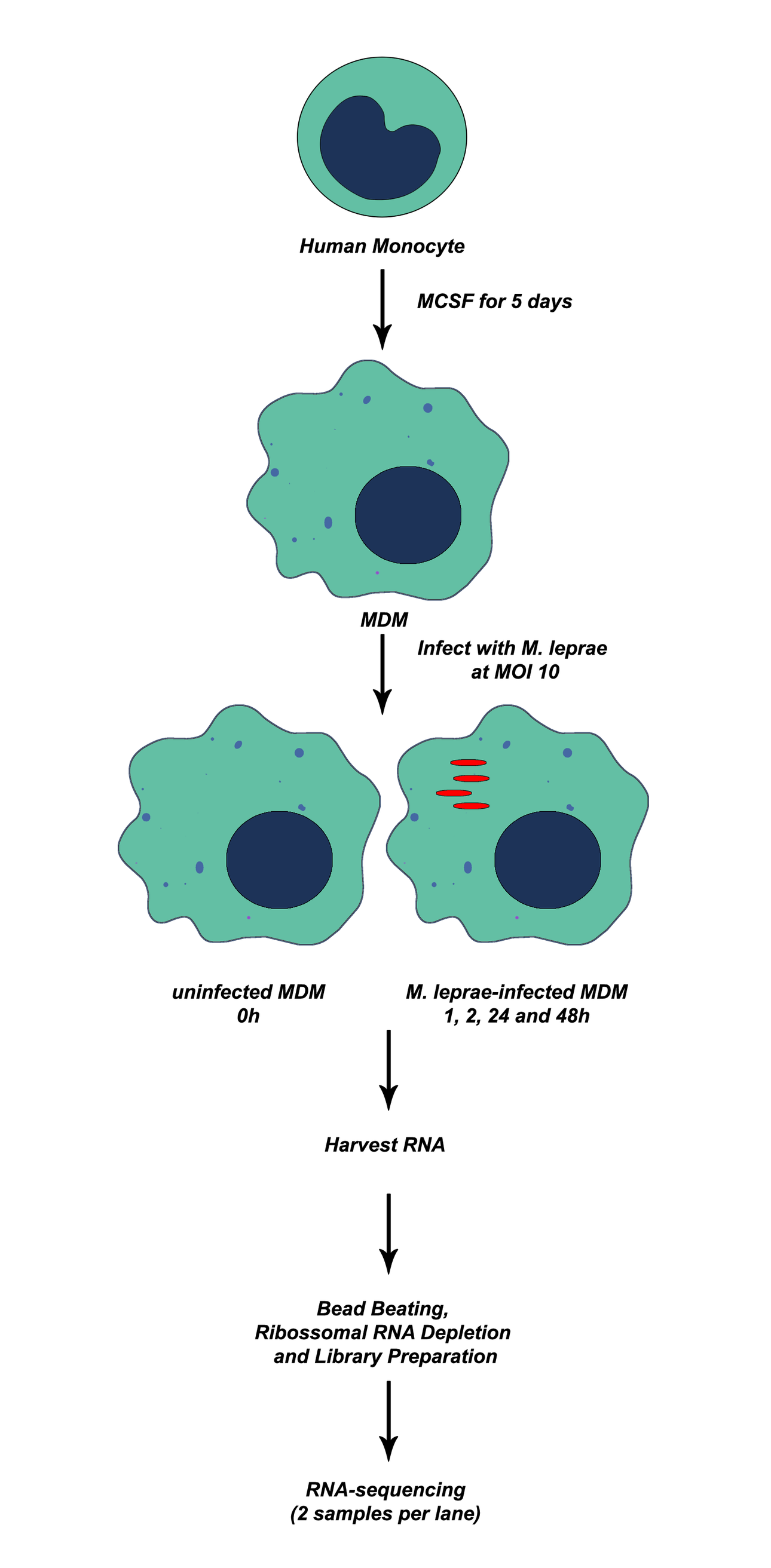

Supplement: S1 Fig — Human monocytes were obtained from a heathy donor and cultured for 5 days with MCSF to differentiate into monocyte-derived macrophages (MDMs). MDMs were infected with live M. leprae and RNA was harvested at 1, 2, 24 and 48h post-infection and prepared for RNA sequencing. Uninfected control is referenced as 0h. (TIF) [file pntd.0007589.s001.tif]

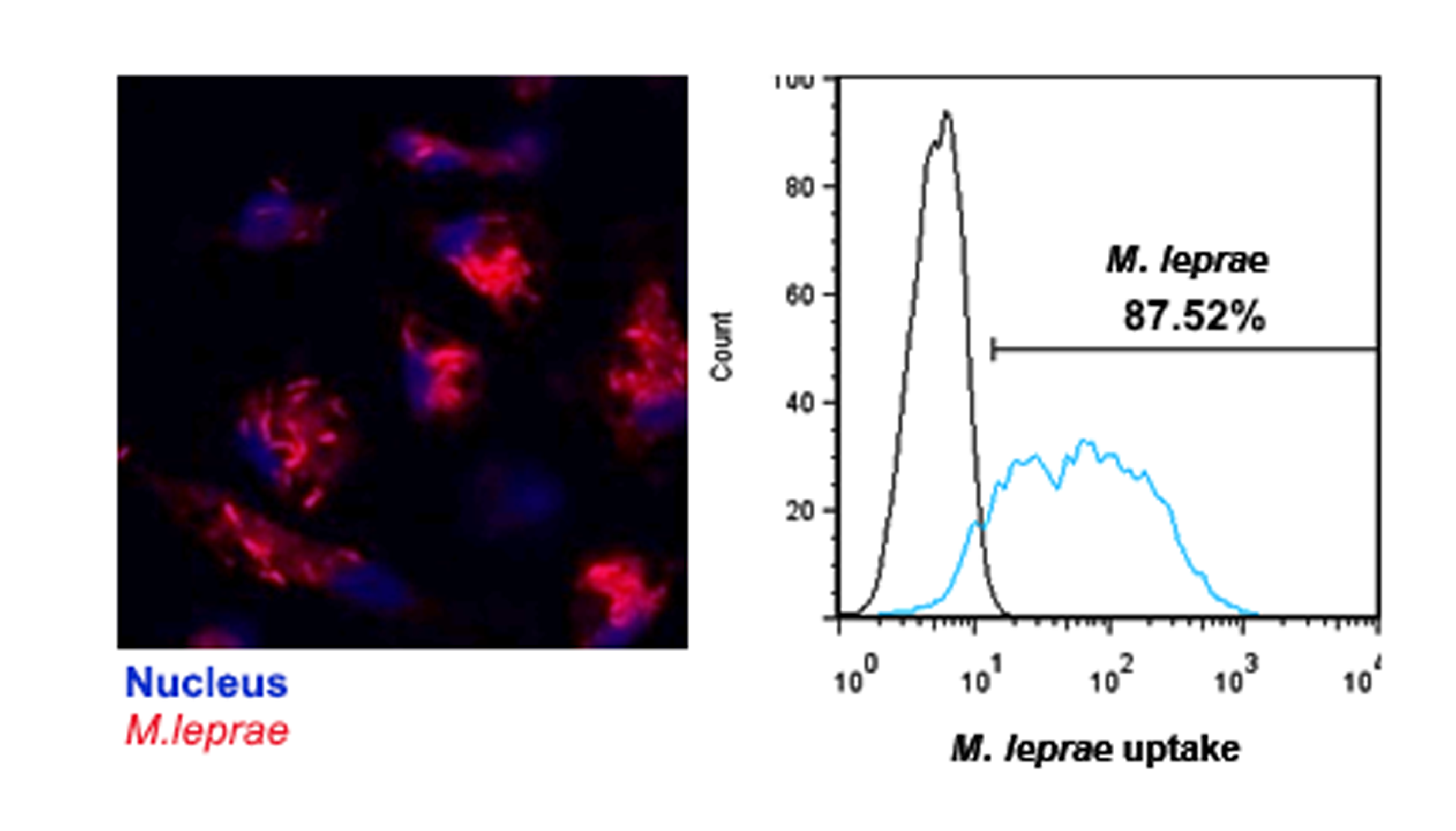

Supplement: S2 Fig — MDMs were infected with PKH26-labeled M. leprae (red) at MOI 10 and uptake was assessed via confocal microscopy (left) and flow cytometry (right). Nuclei are stained with DAPI (blue) (n = 1). (TIF) [file pntd.0007589.s002.tif]

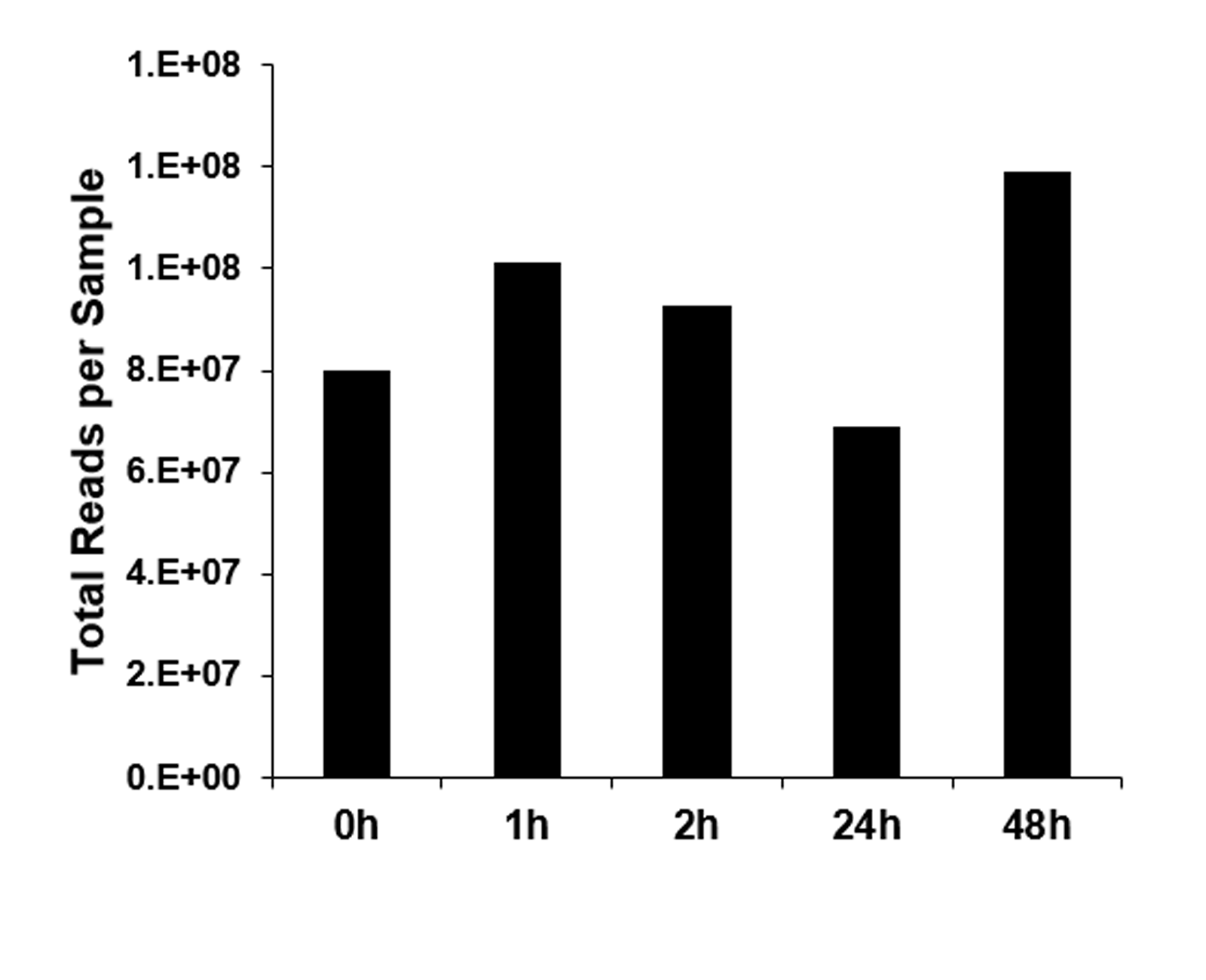

Supplement: S3 Fig — Total number of RNA sequencing reads obtained for the individual sample at 0h (uninfected) and 1h, 2h, 24h and 48h post-M. leprae infection. (TIF) [file pntd.0007589.s003.tif]

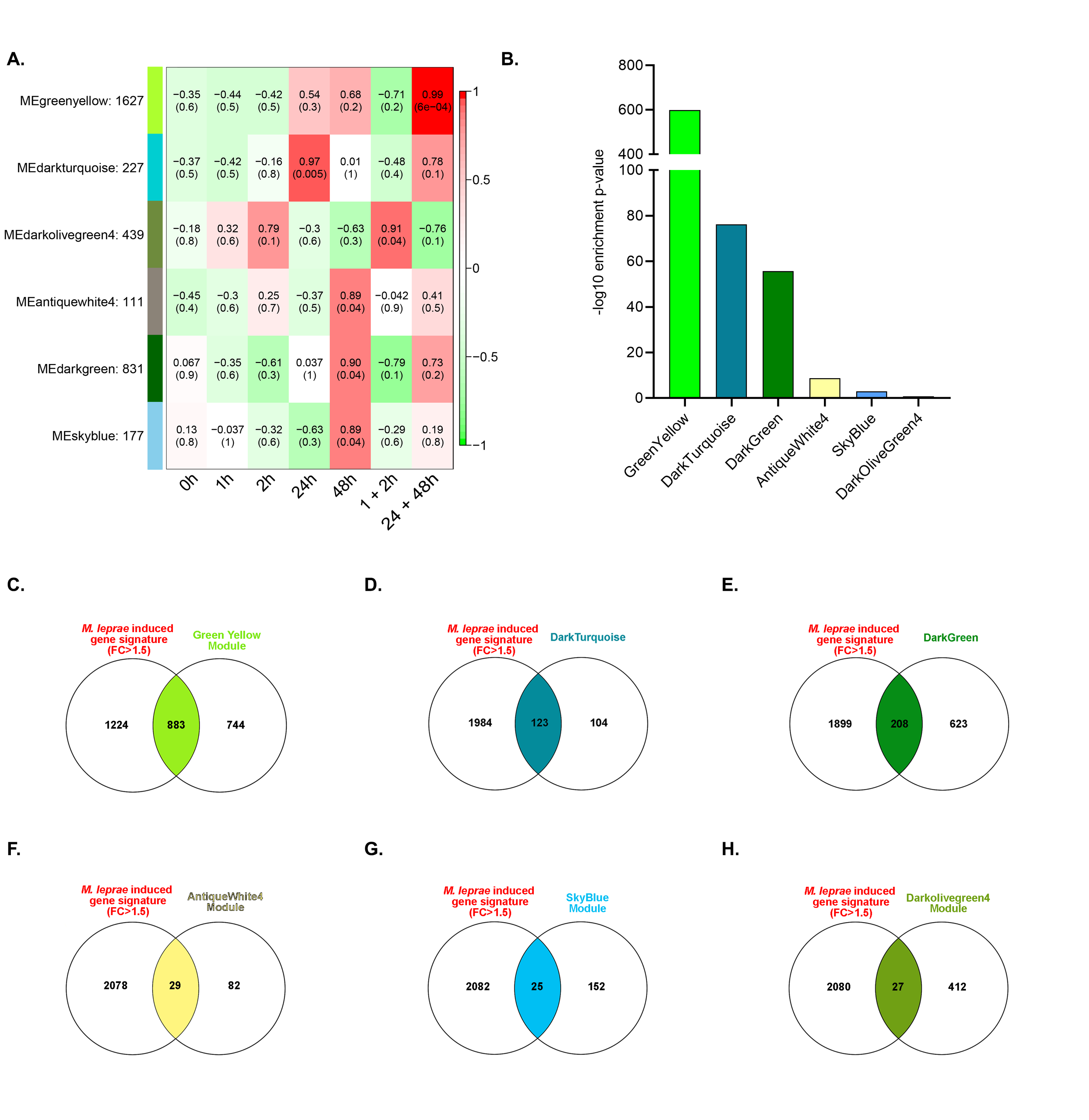

Supplement: S4 Fig — (A). Signed WGCNA of log2RPKM expression values from genes induced by M. leprae at different time points. Correlation of time points (x-axis) to WGCNA module eigengenes (y-axis) are displayed as a heatmap. The p-values (bottom) for each r correlation value (top) are indicated for each module and each time point. Red indicates positive correlation and green indicates an inverse correlation. (B). -log10 enrichment p-value of M. leprae induced genes at 24+48 hours found in the WGCNA modules calculated by hypergeometric test. (C-H). Venn Diagrams depicting overlap between M. leprae induced gene signature and WGCNA modules significantly correlated with infection time points. (TIF) [file pntd.0007589.s004.tif]

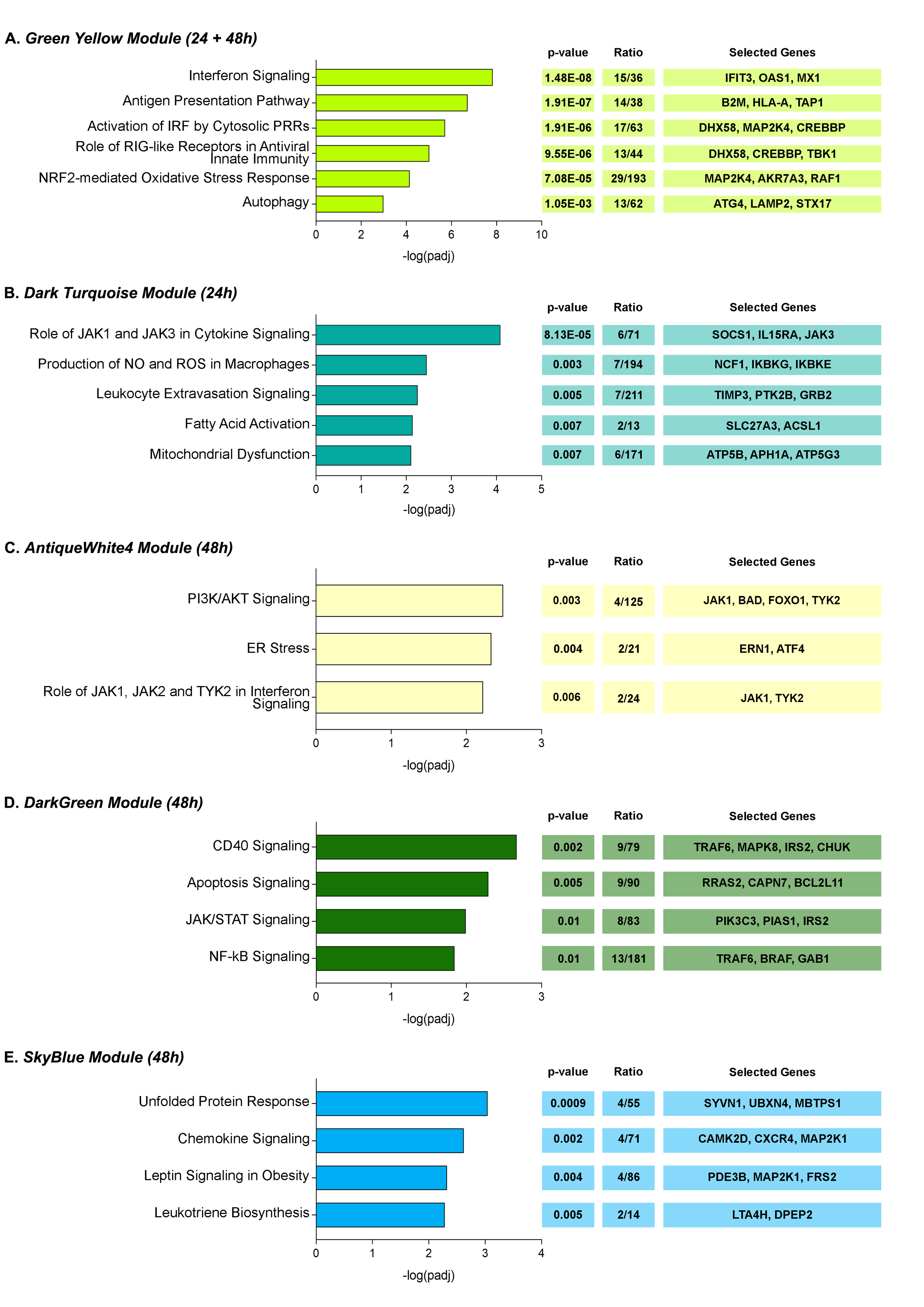

Supplement: S5 Fig — Ingenuity Pathway Analysis (IPA) was performed on the WGCNA modules with a significant positive correlation (r>0.8; p<0.05) with infection time points. (A). ‘GreenYellow’ module correlated with the 24 + 48h vector. (B). ‘Darkturquoise’ module correlated with the 24h time point. (C). ‘AntiqueWhite4’ module correlated with 48h. (D). ‘DarkGreen’ module correlated with the 48h time point. (E). ‘SkyBlue’ module correlated with the 48h time point. IPA core analyses display the Canonical Pathways significantly overrepresented in each module. The p‐value is calculated by Fisher’s Exact Test and measures the significant overlap between the dataset genes and the genes that belong to a canonical pathway in the IPA knowledge database. Adjusted p-values (padj) were calculated using Bonferroni correction. Ratios represent the number of genes in the module that appear in the canonical pathway divided by the total number of genes in that specific canonical pathway. Selected genes of each canonical pathway are displayed based on their functional relevance. (TIF) [file pntd.0007589.s005.tif]

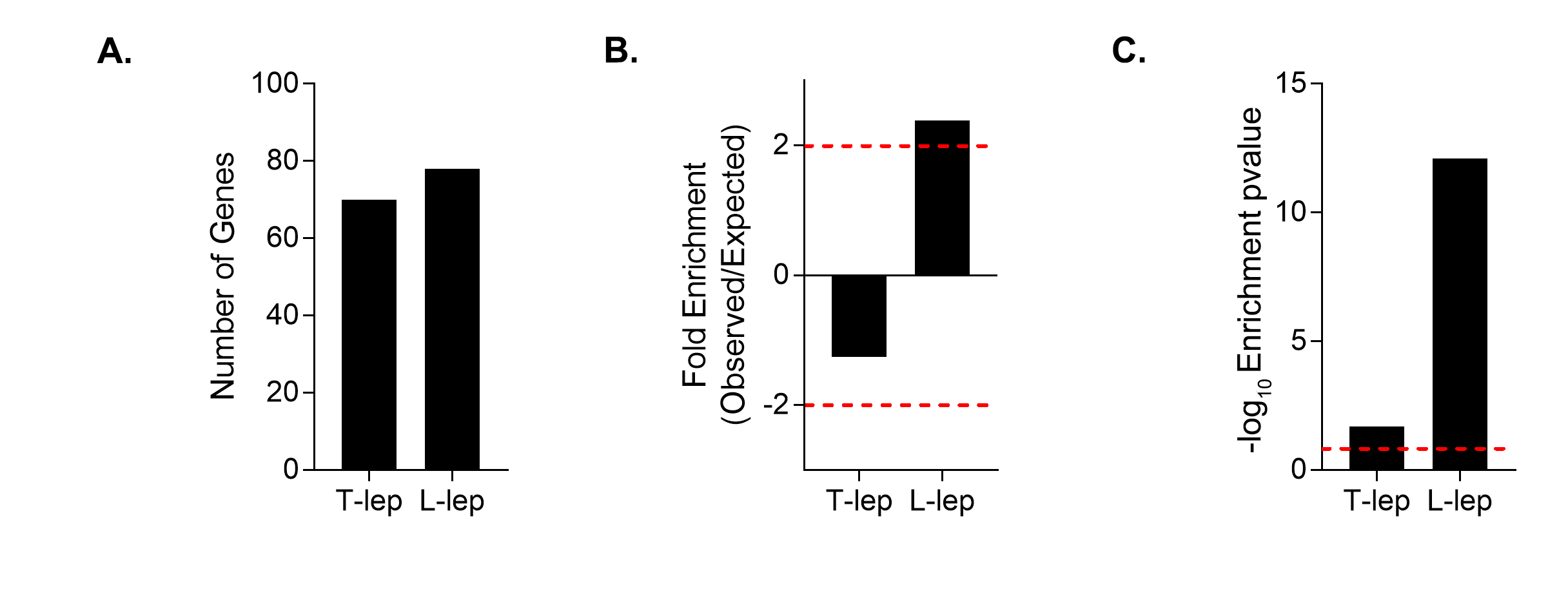

Supplement: S6 Fig — Hypergeometric enrichment analysis of overlap of M. leprae-induced gene signature (fold change >2) with the most expressed genes in L-lep and T-lep lesions (FC>2; p<0.05; probe intensity average>100). (A). Number of genes of the T-lep and L-lep gene signature found in the M. leprae-induced gene signature. (B). Fold change enrichment (see Materials and methods) of T-lep and L-lep genes in the M. leprae-induced gene signature. (C). -log10 enrichment p-value of T-lep and L-lep genes found in the M. leprae induced gene signature calculated by hypergeometric test. (TIF) [file pntd.0007589.s006.tif]
